# Microfluidic High-Migratory Cell Collector Suppressing Artifacts Caused by Microstructures

**DOI:** 10.3390/mi10020116

**Published:** 2019-02-11

**Authors:** Tadashi Ishida, Takuya Shimamoto, Maho Kaminaga, Takahiro Kuchimaru, Shinae Kizaka-Kondoh, Toru Omata

**Affiliations:** 1 Department of Mechanical Engineering, School of Engineering, Tokyo Institute of Technology, Kanagawa 226-8503, Japan; kaminaga.m.ab@m.titech.ac.jp (M.K.); omata.t.aa@m.titech.ac.jp (T.O.); 2 Department of Mechano-Micro Engineering, Interdisciplinary Graduate School of Science and Engineering, Tokyo Institute of Technology, Kanagawa 226-8503, Japan; tokenai.shimamoto@gmail.com; 3 Department of Life Science and Technology, School of Life Science and Technology, Tokyo Institute of Technology, Kanagawa 226-8503, Japan; kuchimaru@jichi.ac.jp (T.K.); skondoh@bio.titech.ac.jp (S.K.-K.)

**Keywords:** high-migratory cell, microfluidic cell collector, balloon, migration assay

## Abstract

The small number of high-migratory cancer cells in a cell population make studies on high-migratory cancer cells difficult. For the development of migration assays for such cancer cells, several microfluidic devices have been developed. However, they measure migration that is influenced by microstructures and they collect not only high-migratory cells, but also surrounding cells. In order to find high-migratory cells in cell populations while suppressing artifacts and to collect these cells while minimizing damages, we developed a microfluidic high-migratory cell collector with the ability to sort cancer cells according to cellular migration and mechanical detachment. High-migratory cancer cells travel further from the starting line when all of the cells are seeded on the same starting line. The high-migratory cells are detached using a stretch of cell adhesive surface using a water-driven balloon actuator. Using this cell collector, we selected high-migratory HeLa cells that migrated about 100 μm in 12 h and collected the cells.

## 1. Introduction

Tumors contain low glucose and low oxygen regions that are over 200 μm away from blood vessels [1]. Some of the cancer cells in this region can migrate long distances, and low oxygen and low glucose conditions promote the migration of cancer cells [2,3]. The high migration ability of some cancer cells is one of the characteristics that cause metastasis at an early stage [4]. Therefore, studies on high-migratory cancer cells are important in understanding cancer metastasis. However, the populations of the high-migratory cancer cells are typically quite low in number, and thus statistical studies on the high-migratory cancer cells are difficult to perform. Furthermore, analytical methods in biochemistry and genetics are difficult due to the small number of high-migratory cancer cells. Protein and gene expression analyses of high-migratory cancer cells are mainly performed using fluorescent microscopy [5]. For these reasons, the selective collection of high-migratory cancer cells is required.

Microfluidic techniques [6] have the following advantages in the development of cellular migration assays: (1) the precise control of cell culture conditions that affect the cellular migration, (2) precise control of cells using flow of culture medium, (3) the ability to separate specific cells from others, and (4) the ability to detach cells using microstructures. As for the control of cell culture conditions, many parameters, such as oxygen, glucose, and temperature, can be controlled using microfluidic devices [7,8,9]. Liquid flow that is caused by microstructures controls cell motion in microchannels and chambers [10,11,12]. Due to this precise control, cells can be sorted by many parameters, such as size, hardness, and molecular expression [13]. Cells can be detached using chemicals and physical/physicochemical methods, such as electric stimulation [14], ultrasonic vibration [15], and transition between hydrophobicity and hydrophilicity [16]. Due to the advantages, many microfluidic devices for migration assay were developed. There are two types of migration assays; one is microscaled scratch assay by preparing cell free areas in the cell confluent condition [17,18,19,20], and the other is multiple chambers that are connected with microchannels [21,22,23]. In the case of microscaled scratch assay, cell free areas in the confluent condition using the following methods: a micropillar contact mask to prevent cells from adhering on the contact area between the micropillars and cell culture surface [17], a membrane to squash cells at the contact area [18], laminar flow of trypsin to release cells from a surface [19], and laminar flow of cell suspension for the local cell seeding [20]. These microfluidic devices for the microscaled scratch assay can visualize cellular migration, but they cannot sort and collect high-migratory cells. In the case of multiple chambers, cells and chemicals are separately introduced into each chamber and they check the chemotaxis through the microchannels [21,22,23]. This type can sort the cells by chemotaxis, but will always receive some influence from microchannels. However, these methods can easily perturb the cell activities and conditions due to the application of signals and restrictions that cells do not usually experience in vivo. For example, microchannels can easily sort high and low migratory cells, because the direction of the migration is regulated to one dimension and, therefore, the migration activity can be easily measured [24,25]. Although the cellular migration is easily measured, the sidewalls of the microchannels affect the cellular migration in terms of motion and speed [26,27]. Furthermore, trypsin solution, which is usually used to detach cells, detaches target cells, as well as cells surrounding target cells due to the diffusion of trypsin. In addition, it may damage the proteins on the surface of the cells [28]. To suppress damages during detachment, other methods have been developed for local detachment of target cells. Patterned poly-N-isopropylacrylamide (pNIPAM) can detach cells [29,30] by utilizing the change of the surface properties from hydrophobic to hydrophilic when the temperature is lowered. Cells that are on gold electrodes with a thiol self-assembled monolayer (SAM) [14] are detached when electrical voltage is applied to the electrodes. These methods can locally detach cells, but the patterned materials form microscopic “steps” at different heights at the edge of the patterns. These steps may affect the migration of cancer cells. A microfluidic device that collects high-migratory cells while suppressing artifacts is required for studies on high-migratory cancer cells.

We developed a microfluidic high-migratory cancer cell collector that sorts cells by their migration ability. This microfluidic device does not have microchannels for cell migration abilities but rather possesses a flat cell culture surface to suppress the artifacts. Cancer cells are locally seeded along the sidewall of a microchamber using laminar flow [31]. The patterned cancer cells randomly migrate at low glucose concentrations. A small number of the cancer cells migrate rapidly and quickly reach the detachment zone, which is separated from the cell-seeded zone. A stretch of cell culture surface using a microactuator that was driven by hydraulic pressure detached the cancer cells. Only the high-migratory cancer cells are detached and then carried downstream, resulting in the collection of high-migratory cancer cells.

## 2. Microfluidic High-Migratory Cell Collector

### 2.1. Concept of the Microfluidic High-Migratory Cell Collector

**Figure 1:**
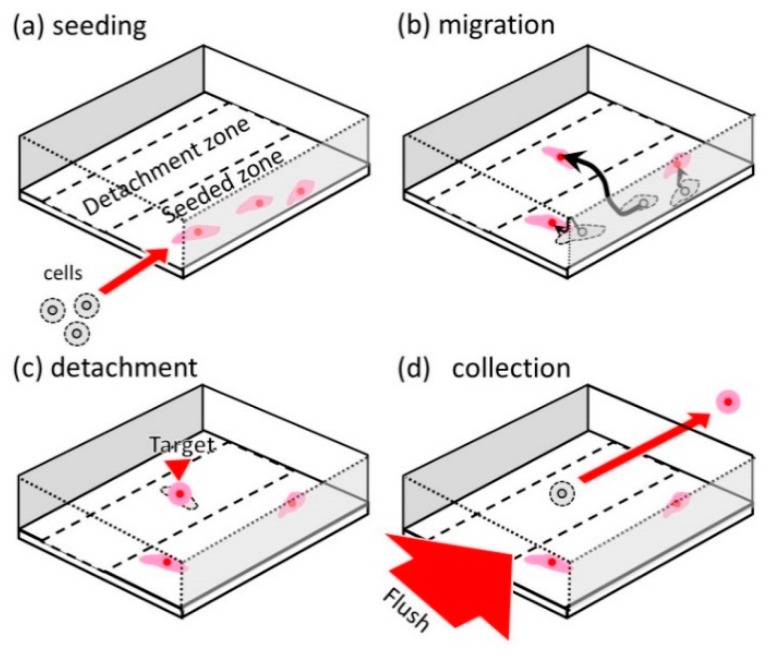
Collection of high-migratory cells. (**a**) Seeding of cells in a cell culture microchamber. (**b**) Cell migration in the cell culture microchamber. (**c**) Detachment of high-migratory cells. (**d**) Collection of the high-migratory cells.

The conceptual illustration of our microfluidic high-migratory cell collector is shown in Figure 1. Cancer cells are seeded in a line pattern along a microchamber wall, which is called the cell-seeded zone (Figure 1a). The seeded cancer cells are cultured and they migrate for certain duration in the microchamber (Figure 1b). Once a small number of the cancer cells reach the cell detachment zone, which is at the distance of 100 μm from the cell-seeded zone, they are detached (Figure 1c)). The detached cancer cells have high-migratory capability relative to other cancer cells, and they are washed away and collected (Figure 1d). 

We developed cell seeding and detachment apparatuses to achieve this concept. The cell seeding apparatus seeds cancer cells in a line pattern using the laminar flow between cancer cell suspension and cell culture medium. The cell detachment apparatus applies shear stress to the hemidesmosomes between cancer cells and the extracellular matrix to achieve detachment [32]. The shear stress is caused by the mechanical stretch of the cell’s migrating surface due to the expansion of the balloon under this surface, which is usually used for the mechanical stimuli to measure the cellular responses [33,34,35].

### 2.2. Design of the High-Migratory Cell Collector

We designed the microfluidic high-migratory cell collector by combining the cell seeding and detachment apparatuses. For cell seeding, laminar flow is generated using a cell suspension and cell culture medium. The flow rate of the cell suspension is low when compared to that of the cell culture medium, such that the cancer cells are seeded in a line pattern along the wall of a cell culture microchamber. For cell detachment, a water-driven balloon underneath the cell migrating surface is utilized (the area upon the balloon is the cell detachment zone). When the balloon is expanded, the hemidesmosomes between the cancer cells and the extracellular matrix are sheared, resulting in their breakages. The water-driven balloon mechanically detaches the adherent cancer cells and thus the detachment function does not use chemicals or unfamiliar signals to the cells. Although this kind of balloon is usually actuated by compressed air, we used water instead. This is because water can precisely control the motion of a balloon in volume, which prevents the balloon from irregular expansion and blowout. The locations of the cell-seeded zone and the cell detachment zone give the cells the same start and goal lines. The distance between the cell-seeded zone and the cell detachment zone sorts the high-migratory cancer cells from the entire population of seeded cancer cells, because low-migratory cancer cells cannot reach the cell detachment zone earlier than the high-migratory ones. The distance between the cell-seeded zone and the cell detachment zone is set to the relatively short average travel distance of the high-migratory cells. This is a key factor in sorting cancer cells according to their migration capability without using any microstructures that could cause artifacts.

**Figure 2:**
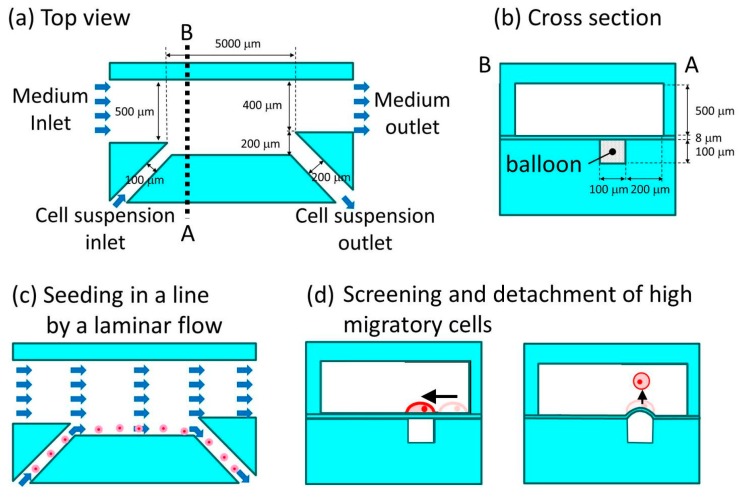
Microfluidic high-migratory cell collector. (**a**) Top view and (**b**) cross section of the microfluidic device and its specifications. (**c**) Seeding cancer cells in a line pattern by laminar flow. (**d**) Screening for high-migratory cancer cells, and detachment of the cells by the expansion of a balloon.

To achieve the aforementioned functions, we designed a high-migratory cell collector (Figure 2). The device has two inlets and two outlets for the laminar flow (Figure 2a). The cell culture medium inlet and cell suspension inlet are 500 μm and 100 μm in width, respectively. Conversely, the cell culture medium outlet and cell suspension outlet are 400 μm and 200 μm in width, respectively. The dimensions of the inlets and outlets were designed by trial and error in order to obtain the narrowest laminar flow of the cell suspension for the narrow line pattern of cells. In order to screen as many cells as possible, the device was designed such that the length of the cell culture microchamber is 5000 μm, because the number of cells that can be linearly aligned against a given length of microchamber wall is limited. For the implementation of the balloon, the device consists of three layers: a cell migration layer, a membrane layer, and a balloon layer (Figure 2b). The membrane is 8 μm in thickness and it is coated with collagen. The balloon is a dead-end microchannel, 5000 μm in length, which aligns itself in parallel to the wall of the cell culture microchamber. The balloon is located 200 μm from the wall of the cell culture channel. This is because the width of the cell-seeded zone is around 100 μm due to the laminar flow, and the top 10% of HeLa cells (based on migration capability) travel over 90 μm in 12 h at a high glucose concentration (high-migratory cancer cells) (Figure 2c) [3]. The adhesive cancer cells travel on the surface that is coated by collagen. They are detached in the cell detachment zone by the expansion of the balloon (Figure 2d), and they are carried forward by the laminar flow, resulting in their entry into the medium outlet.

### 2.3. Fabrication Process of the Microfluidic High-Migratory Cell Collector

**Figure 3:**
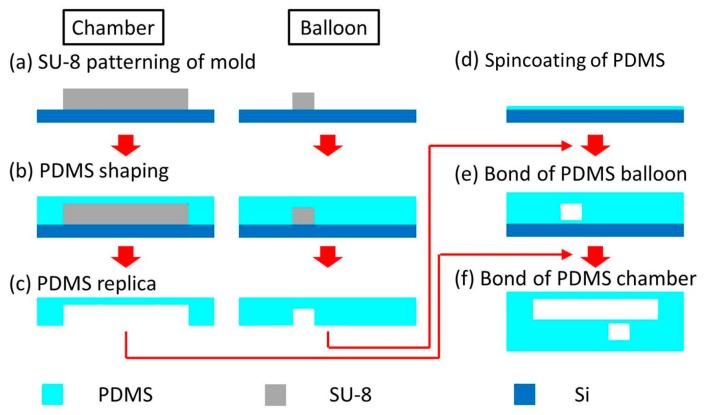
Fabrication process of a microfluidic high-migratory cell collector. (**a**) SU-8 patterning of the molds for a balloon and a microchamber. (**b**) polydimethylsiloxane (PDMS) shaping of a balloon and a cell culture microchamber. (**c**) PDMS replica of a balloon and a microchamber. (**d**) Spincoating of PDMS. (**e**) Bond of a thin membrane to a PDMS balloon. (**f**) Bond of the PDMS structure made in (e) to a PDMS cell culture microchamber.

The fabrication process of the microfluidic high-migratory cell collector is shown in Figure 3. The polydimethylsiloxane (PDMS) structures were fabricated by soft lithography [36]. SU-8 (Microchem) was patterned on a silicon substrate. SU-8 2150 (490 μm thick) and SU-8 3025 (80 μm thick) were used for the microchamber and balloon molds, respectively (Figure 3a). PDMS (Silpot 184W/C, Daw Corning Toray, Tokyo, Japan) was poured into the molds. The ratio of base to crosslinker was 10:1. The molds were cured for shaping (Figure 3b) and then peeled off, resulting in PDMS replicas. (Figure 3c). A thin PDMS membrane was formed by spincoating and then cured (Figure 3d). Before peeling off the membrane, the PDMS structure for balloons was bonded onto the membrane by irradiation with vacuum ultraviolet light (VUV. 192 nm in wavelength) (Figure 3e) [37]. The bonded PDMS structure was then peeled off. The bonded PDMS structure and the PDMS structure for the cell culture microchamber were also bonded by irradiation with VUV (Figure 3f). The cell culture microchamber was coated with collagen (Cellmatrix Type IC, Nitta Gelatin).

**Figure 4:**
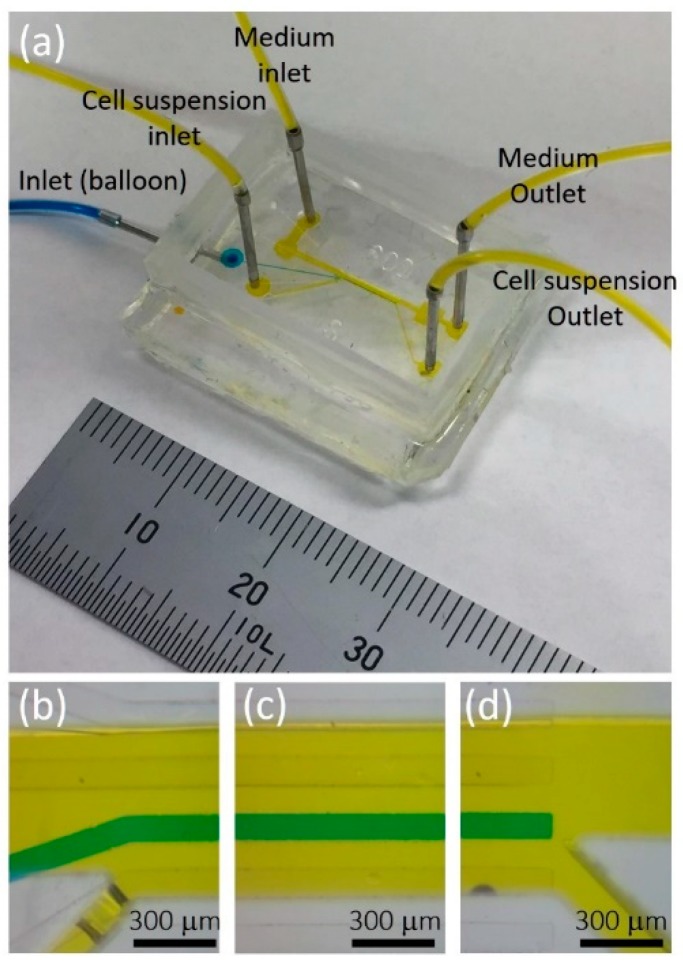
Fabricated microfluidic high-migratory cell collector. (**a**) Overview of the device. Magnified images of (**b**) inlets of culture medium and cell suspension. (**c**) Cell culture microchamber where high-migratory cancer cells are selected. (**d**) Outlets of culture medium and cell suspension.

Figure 4 shows the fabricated microfluidic high-migratory cell collector. It is filled with dye-containing water for visibility. Yellow-dyed water is introduced to the cell culture microchamber through the cell culture medium and cell suspension inlets and outlets. Blue dyed water is introduced to the water-driven balloon. (Figure 4b–d).

### 2.4. Experimental Setup

The microfluidic high-migratory cell collector was placed in a homemade incubation box to control the atmospheric composition, temperature, and humidity. A gas line (air:CO_2_ = 100:6) maintained the CO_2_ concentration at 5.2%. Using a heater and a thermocouple, the temperature was maintained at 37 °C. Humidity was maintained over 80% to mimic the conditions inside a typical CO_2_ incubator. A syringe pump (KD-200, KD scientific, Holliston, MA, USA) was connected to the microfluidic high-migratory cell collector for the introduction of the cell culture medium, which consisted of Dulbecco’s Modified Eagle Medium (DMEM, D-MEM with L-glutamine, phenol red, and sodium pyruvate; Wako) containing fetal bovine serum (FBS, regular; Wako) that was inactivated at 56 °C for 30 min, penicillin, and streptomycin (PS, Penicillin-Streptomycin solution 100X, Wako) in the following proportions: 10% FBS, 100 units/mL penicillin, and 100 mg/mL streptomycin. We prepared two cell culture media (mixture of DMEM and FBS), one containing a high glucose concentration (25.7 mM) for cell culture, and one containing a low glucose concentration (0.7 mM) for cell migration. The cell culture medium was introduced into the microfluidic high-migratory cell collector at 5 μL/min, when the cells were cultured. The cells inside the microfluidic high-migratory cell collector were observed using an inverted optical microscope (IX-73, Olympus, Tokyo, Japan). They were recorded with time lapse imaging while using a charge-coupled device (CCD) camera (STC-TC202USB-AS, SENTECH). We used the HeLa cell line, which was obtained from ATCC (Manassas, VA, USA), and it is one of the most widely used human cell lines derived from human cervical cancer cells. 

## 3. Results & Discussion

### 3.1. Cell Seeding Experiment for a Line Pattern

**Figure 5:**
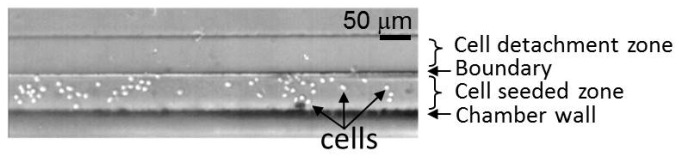
Cells seeded in a line pattern using the laminar flow.

**Figure 6:**
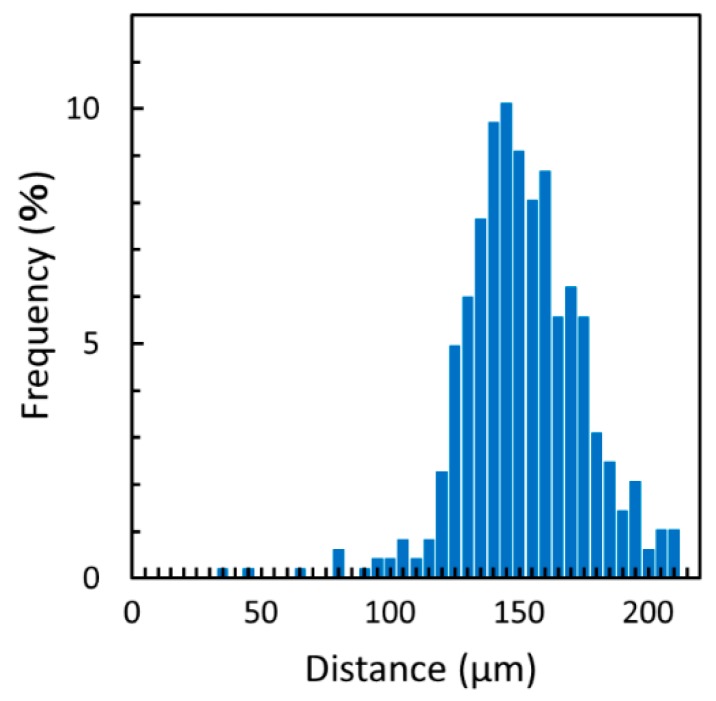
Cellular distribution seeded by the cell patterning method using laminar flow. The distance is the length between the edge of the cell detachment zone and each seeded cell.

The laminar flow between the cell culture medium and the cell suspension was used for cell seeding along the wall of the cell culture microchamber (Figure 5). The white dots represent cells. The flow rates of the cell culture medium and cell suspension were 4 μL/min and 0.5 μL/min, respectively. The density of the cell suspension was 5.0 × 10^6^ cells/mL. Figure 6 shows the distribution of the seeded cells from the wall of the cell culture microchamber. The average distance between the balloon and the seeded cells was 149 ± 23 μm (n = 484). 99% of cells were located over 90 μm from the edge of the balloon.

### 3.2. Water-Driven Balloon for Cell Detachment

**Figure 7:**
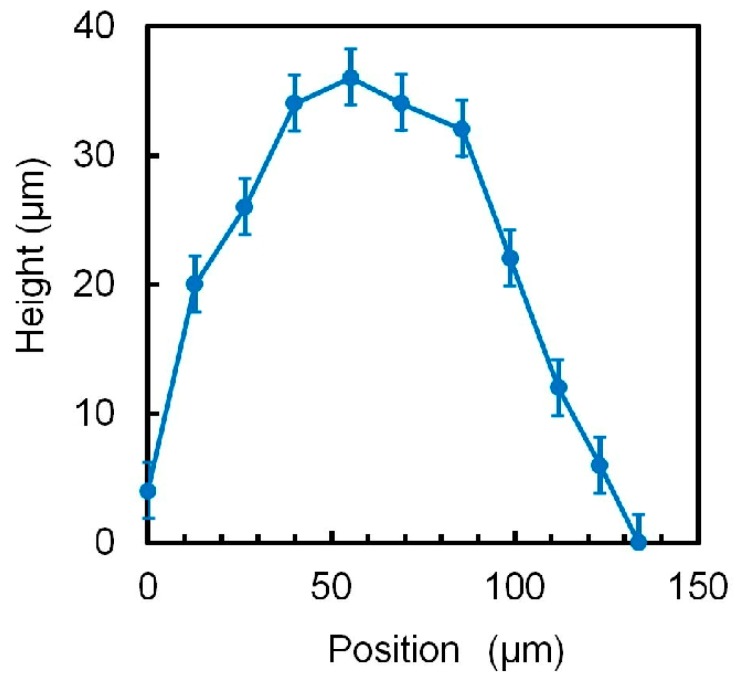
Surface profile of the expanded balloon. Error bar is accuracy of our measurement method.

The water-driven balloon was expanded by introducing water. The balloon was initially filled with water, such that the surface profile was flat (flat condition). The surface profile of the balloon changed (Figure 7) when 300 μL of water was introduced (expanded condition). The length of the surface of the expanded balloon was 169 μm. The surface profile was measured by changing the position of an objective lens of the microscope. When considering that the length of the balloon in the flat condition was 100 μm, the elongation ratio was 69%. The motion of the balloon from the flat to expanded condition was completed in 170 ms, and vice versa. We did not experience a burst of the balloon after tens of the cyclic motions between the flat and expanded conditions.

**Figure 8:**
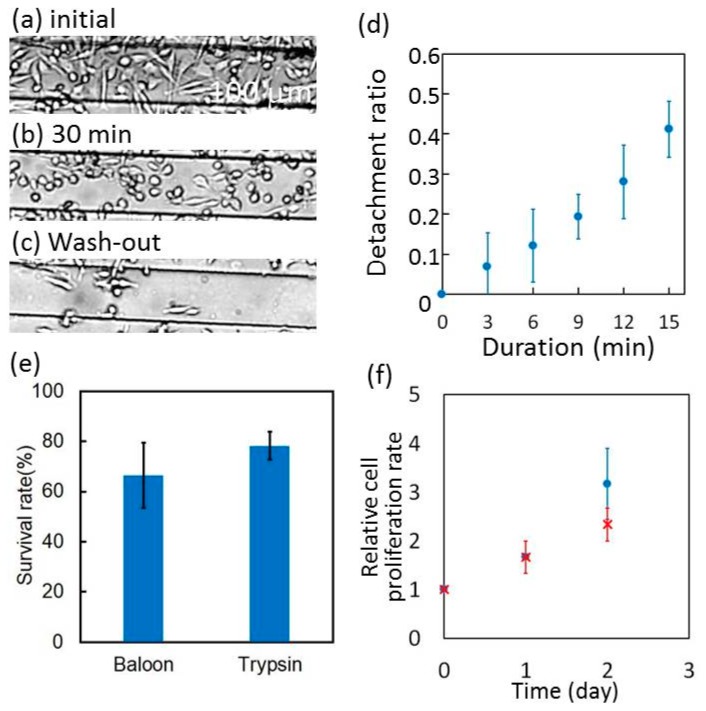
Cell detachment using balloon expansion. (**a**) Adhesive cells on balloon. (**b**) Detachment with the expansion of the balloon for 30 min in total. (**c**) Cells remaining after wash-out. (**d**) Cell detachment ratio as a function of duration of balloon expansion (n = 3. Error bar is standard deviation.). (**e**) Comparison of survival rate between cells detached by either the balloon or trypsin (n = 3. Error bar is standard deviation.). (**f**) Comparison of relative cell proliferation rates between cells detached by either the balloon or trypsin (n = 3. Error bar is standard deviation.).

Using the balloon, we detached the adhesive cells on the surface of the balloon. Initially, the cells that were cultured on the surface of the balloon were confluent (Figure 8a). The balloon was intermittently expanded by 40% and then flattened. The cycle between the expanded condition for 3 min and the flat condition for 1 min was repeated. The ratio of the detached cells to the total cells (cell detachment ratio) was plotted as a function of total duration of the balloon expansion. In this analysis, we defined that cells of circular shape without any pseudopodium structures were detached (Figure 8b). The initial cell detachment ratio was 0. In the first 15 min, it increased at a rate of 0.027 min^−1^ and then reached 0.41 after 15 min (Figure 8d). The cells continued to detach due to the expansion of the balloon for 30 min (Figure 8b) and were then flushed away. The cell detachment ratio finally reached 0.8 (Figure 8c). Most of the remaining cells were aligned in parallel with the balloon. This is because the expansion ratio parallel to the balloon was smaller than that perpendicular to the balloon. The damage of the detached cells by this method was tested using 0.4 w/v% trypan blue solution just after the detachment. The survival rates of the balloon method and trypsin were comparable (Figure 8e). The cells that were detached by this method were cultured in the cell culture microchamber under 38 °C and 5% CO_2_ conditions. After 2 h, 97% of the detached cells adhered on the surface of the balloon again. The relative cell proliferation rate of the cells detached by the balloon was not significantly different from those that were detached by trypsin treatment (Figure 8f). 

### 3.3. Collection Experiment of High-Migratory Cancer Cells

**Figure 9:**
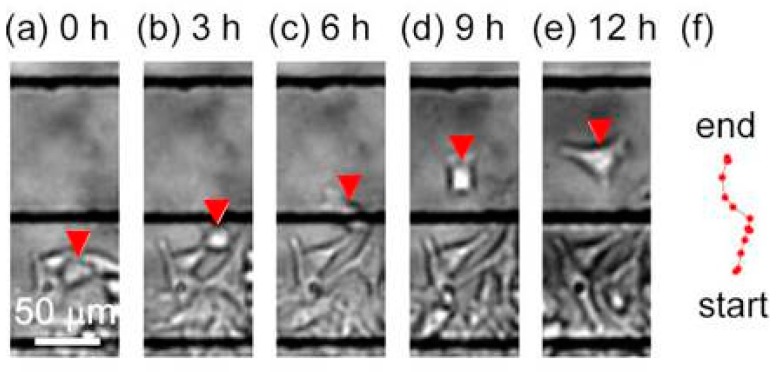
Screening for a high-migratory cell. (**a**) 0 h, (**b**) 3 h, (**c**) 6 h, (**d**) 9 h, and (**e**) 12 h, (**f**) the trajectory of the high-migratory cell traced every hour.

The microfluidic high-migratory cell collector was tested. Before the migration assay, cells were seeded in a line pattern using laminar flow and they were cultured at a high glucose concentration of 25.2 mM for 2 h. They adhered to the cell culture microchamber. The glucose concentration was switched to low glucose concentration to promote migration. One of the cells significantly migrated for 12 h (Figure 9a–e). According to the trajectory of the high-migratory cell, the travel length was 109 μm and the distance between the start and end points was 90 μm, perpendicular to the direction of flow in the cell culture microchamber (Figure 9f). The high-migratory cell reached the cell detachment zone. 

**Figure 10:**
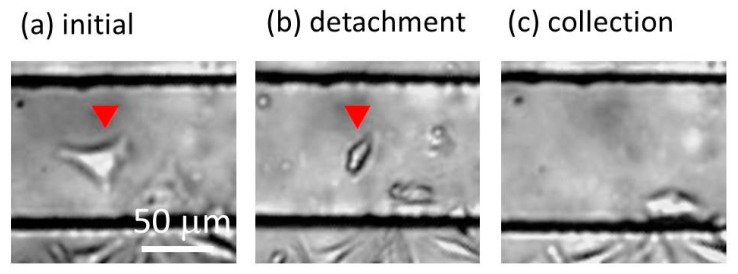
Detachment and collection of a high-migratory cell. (**a**) Initial condition. (**b**) The adhesive cell after the expansion of balloon for 20 min. It was detached. (**c**) Collection of the detached cell by flowing the cell culture medium at 500 μL/min.

The high-migratory cell was detached by the expansion of the water-driven balloon, just after the arrival at the cell detachment zone of the high-migratory cells. The high-migratory cell initially adhered on the balloon surface (Figure 10a). The balloon was kept in its expanded state for 20 min. The high-migratory cell was detached (Figure 10b). The flow rate of the cell culture medium was increased by 100 μL/min for the collection of the cancer cell. The detached cell was carried downstream at 500 μL/min (Figure 10c). With this method, the high-migratory cell was selectively collected. For the analysis of cells, we have to repeat this experiment to increase the number of collected cancer cells with high migration capability.

Authors should discuss the results and how they can be interpreted in the perspective of previous studies and of the working hypotheses. The findings and their implications should be discussed in the broadest context possible. Future research directions may also be highlighted.

## 4. Conclusions

We developed a microfluidic high-migratory cell collector to separate high-migratory cells from other cells. In this device, the cells patterned in a line to set the same start line for the migration assay. High-migratory cancer cells were screened based on their migration capability without using microchannels, resulting in the minimization of artifacts to their migration capability. The balloon that was expanded by water introduction selectively detached the high-migratory cancer cells, which were then collected. This operation has to be repeated, resulting in the collection of a sufficient number of high-migratory cancer cells for the biological and biochemical analyses. In the future, the shape of the seeding zone will be modified to collect more cells in a single experiment.
